# Broad spectrum antimicrobial PDMS-based biomaterial for catheter fabrication

**DOI:** 10.1186/s40824-021-00235-5

**Published:** 2021-10-21

**Authors:** Arunmozhiarasi Armugam, Siew Ping Teong, Diane S. W. Lim, Shook Pui Chan, Guangshun Yi, Dionis S. Yew, Cyrus W. Beh, Yugen Zhang

**Affiliations:** 1 Institute of Bioengineering and Bioimaging, 31 Biopolis Way, The Nanos, Singapore, 138669 Singapore; 2 Molecular Engineering Lab, Institute of Molecular and Cell Biology, 61 Biopolis Drive, The Proteos, Singapore, 138673 Singapore

**Keywords:** Antimicrobial, Composite, PDMS, Catheter, Biomaterial

## Abstract

**Background:**

In addition to the widespread use of antibiotics in healthcare settings, the current COVID-19 pandemic has escalated the emergence of antibiotic resistance. Nosocomial infections among hospitalized patients is a leading site for such resistant microbial colonization due to prolonged use of invasive devices and antibiotics in therapies. Invasive medical devices, especially catheters, are prone to infections that could accelerate the development of resistant microbes. Often, catheters - particularly urinary catheters - are prone to high infection rates. Antibiotic-coated catheters can reduce infection rates and although commercially available, are limited in efficacy and choices.

**Methods:**

Herein, a novel and facile method to fabricate **PMDS**-based biomaterial for the development of antimicrobial eluting catheters is presented. Silicone based organic polymer polydimethylsiloxane (**PDMS**) was used to prepare a biomaterial containing novel polymeric imidazolium antimicrobial compound.

**Results:**

It was found that the **PDMS**-based biomaterials could eradicate microbial colonization even after 60 days in culture with continuous microbial challenge, be recycled over multiple uses, stored at room temperature for long-term usage and importantly is biocompatible.

**Conclusion:**

The **PDMS**-based biomaterial displayed biocidal functionality on microbes of clinical origin, which form major threats in hospital acquired infections.

**Graphical Abstract:**

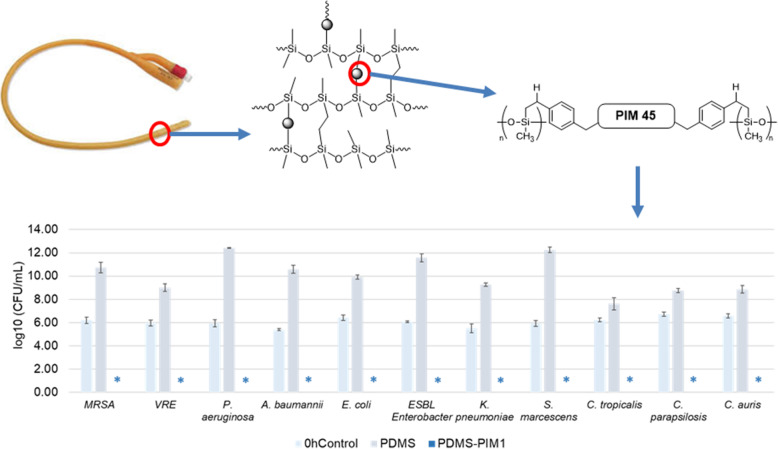

**Supplementary Information:**

The online version contains supplementary material available at 10.1186/s40824-021-00235-5.

## Introduction

The COVID-19 pandemic has introduced another wave of inappropriate and excessive utilisation of antibiotics, biocides, and disinfectants that will hamper ongoing worldwide efforts towards the control of antimicrobial resistance. Nosocomial infections have escalated to a new magnitude of emergency in the current pandemic era [1, 2] at a time when medical device-associated infections have superseded surgical site infections in healthcare services [3]. As part of this trend, urinary catheter associated infections are becoming increasingly rampant in healthcare settings with high mortality and morbidity events [4, 5]. To circumvent device-related infection, several antimicrobial-coated catheters (silver-coated Dover™ silicone catheter and Rochester Medical Magic 3 nitrofurazone-coated silicone catheter) have emerged in clinical use, albeit with limited success. The existing products, particularly those relying on metal ions as the antimicrobial component, are subject to the following concerns: (a) limited efficacy; (b) inability to sustain continuous antimicrobial functionality over a prolonged period [6–8] as well as (c) potential toxicity [9]. Thus, there is a pressing need to explore the fabrication of new biomaterials with antimicrobial properties that could address these disadvantages.

In order to overcome infections related to medical devices, materials for medical devices, especially catheters, are fabricated to contain inherent antimicrobial properties. Two of the widely reported methods for achieving this are surface grafting using functionalized groups and surface coating using antimicrobial compounds [4, 10]. Though showing encouraging results in the laboratory, materials prepared by surface grafting/functionalization [11] and surface coating [12] procedures often deplete their antimicrobial functionality and efficiency within a short duration. This was observed for the commercially available products. Several studies have also reported on the feasibility of impregnating antimicrobial compounds within the catheter material using the swelling to evaporation method. Peritoneal silicone catheters impregnated with rifampicin, triclosan and trimethoprim inhibited colonization of Methicillin-resistant *Staphylococcus aureus* (***MRSA***) for 90 days in a flow model [13]. The authors proposed that the combinatorial antimicrobial impregnated catheter could prove beneficial for long-term clinical applications [14]. Recently, casting a blend of biomaterials with the antimicrobial agents to produce composite biomaterials has been demonstrated [15]. While surfaces that were functionalized, grafted or coated with antimicrobials were observed to only be bacteriostatic, biomaterials that were impregnated or casted with antimicrobials appear to exhibit bactericidal properties [16].

Though various polymers are being used to fabricate a wide range of medical devices, polydimethylsiloxane (**PDMS**) elastomers have emerged as a promising lead for catheter development. **PDMS** is innately biocompatible, chemically stable, transparent and mechanically elastic. Nevertheless, being chemically inert, **PDMS** lacks reactive functional groups on its surface. Pre-treatments such as high-energy treatment or chemical etching are usually required to incorporate useful functional groups for surface modification [16]. On the other hand, impregnation of useful antimicrobials or biocides into commercially available silicone catheters is heavily dependent on the solubility of the antimicrobial compounds in organic solvents [14, 17, 18]. In both cases, the entire process is usually complex and laborious, requiring several sequential stages to remove the solvents used for grafting or impregnation. These laborious protocols are unfavourable and hinder their application in industrial-scale fabrications. In addition, these biomaterials have short shelf lives (durability) and short-lived antimicrobial efficacy. They also require low temperature storage conditions, adding to their production cost.

Herein, a facile and simple method for fabricating **PDMS**-based biomaterials (**PDMS-PIM**) exhibiting antimicrobial properties, is reported. Instead of grafting or functionalizing the **PDMS** surface, we modified a novel amphiphilic main-chain poly-imidazolium (**PIM**) [19, 20] with a styrene functional groups that react directly with **PDMS** precursors and integrate to form a functional biomaterial. It is noteworthy that **PDMS** biomaterials are also used in biomedical applications as medical implants in orthopaedic, dental, cardiovascular, prostheses and as well as in tissue engineering [21]. The release profile indicated an initial burst of antimicrobial compound (PIM), followed by sustained release. Biocidal properties of **PDMS-PIM** were observed over duration up to 60 days under continuous microbial challenge. In addition, excellent biocidal effect was also demonstrated against several multi drug resistant (MDR) clinical isolates, illustrated the broad spectrum antimicrobial property of the **PDMS-PIM** biomaterial. The biocidal properties of our biomaterial was observed up to 6 months upon storage at room temperature, thus showing a promising, long shelf life.

## Materials and methods

### Microbial strains and cell lines

Microbes, *Escherichia coli* (*E. coli*, ATCC 15036), *Staphylococcus aureus* (*S. aureus*, ATCC 6538), *Candida albicans* (*C. albicans*, ATCC 10231) and murine fibroblast cell line (NCTC clone 929; L929, ATCC® CCL1™) were purchased from American Type Culture Collection (ATCC.org). *Klebsiella pneumonia* (CI0027), *Serratia marcescens* (CI0183A1), *Pseudomonas aeruginosa* (CI0183A2), *Acinetobacter baumannii* (RI0139A), *Enterobacter aerogenes* ESBL-sensitive (RI0006A2) *Methicillin-resistant Staphylococcus aureus* (R10309N), *Vancomycin resistant Enterococcus faecium* (RI0252N1), *Candida tropicalis* (RI0243A), *Candida parapsilosis* (ATCC 22019) *Candida auris* (NCPF 8977) were kind gift of Dr. Shawn Vasoo from National Centre for Infectious Disease, Singapore.

### Preparation of PDMS silicone rubber

**PDMS** Sylgard®R 184 (Dow Corning Corporation) is a heat curable **PDMS** supplied as a two-part kit consisting of pre-polymer (base) and cross-linker (curing agent) components. The pre-polymer and cross-linker were mixed at a 10:1 weight ratio and cast onto a petri dish (manufacturer’s recommendation). The composite was degassed under vacuum to remove air bubbles and processed for curing at 70 °C overnight. The cured **PDMS** sheet was cut into square pieces of 2.5 cm × 2.5 cm, with thickness around 1 mm. The **PDMS** composite materials were then washed with ethanol and dried at 60 °C before use.

### Synthesis of PIM45-vinyl imidazolium antimicrobial polymer

The synthesis of **PIM45-vinyl** was carried out between **PIM45** with imidazole terminal and 4-vinylbenzyl chloride. The imidazolium polymer material, **PIM45** was synthesized using the previously reported method [19]. 4-vinylbenzyl chloride (56 mg, 3.0 eq of **PIM45**) was added to 1.25 mL EtOH solution containing **PIM45** (250 mg, 0.122 mmol). The mixture (in a sealed vial) was stirred at 65 °C for 16 h. The suspension was transferred to a 15 mL centrifuge tube and purified following repetitive precipitation from THF. The compound was then dried under reduced pressure at 90 °C that resulted in a pale yellow powder of **PIM45-vinyl** as end product. The **PIM45-vinyl** synthesis scheme is presented in Fig. S1 and the synthesised derivative was verified by proton nuclear magnetic resonance (^1^H NMR) spectrum as shown in Fig. S5.

### Preparation of **PIM45-vinyl** casted composite **PDMS-PIM** material.

**PIM45-vinyl** (0.11 g, 1 wt%) in 2 ml THF was mixed thoroughly with **PDMS** base and curing agent (10:1 ratio) in a mix-cure process. The quasi-solid substance were degassed under vacuum to remove solvent and air bubbles followed by curing at 70 °C (overnight). The cured **PDMS** sheet was cut into square pieces of 2.5 cm × 2.5 cm, with thickness around 1 mm. The **PDMS** samples were rinsed with ethanol and dried at 60 °C before use.

### Characterization of biomaterial surface

- Surface wettability measurement.

**PDMS** based silicone materials are naturally hydrophobic. The static contact angle of **PDMS-PIM** and pristine **PDMS** material surfaces were measured on OCA15 contact angle analyzer (Future Digital Scientific Corp., U.S.A.). Deionized (DI) water (2 μL) at 3 μL/sec, was used for all measurements [17]. All samples were analysed in triplicates, and the static contact angle data were presented as mean ± SD. **PIM45-vinyl** casted composite **PDMS** material at various weight % of **PIM45-vinyl** was selected for our study and depicted as **PDMS-PIMx** (where x represents the % **PIM45-vinyl** used to formed the composite).

b) Surface roughness assessment.

Topographic images of the **PDMS** and **PDMS-PIM** material surfaces were obtained from Nanoscope 9.7 Dimension ICON Atomic Force Microscope (Bruker). **PDMS** material were rinsed in deionised water and 70% ethanol before drying them at 60 °C. The dried material was subjected to a jet of compressed air to remove any particles on the surface. AFM tapping mode (phase contrast) measurements were performed with a scanning rate of 0.995 Hz using a non-contact long reflex (NCLR) probe (k = 48 N/m, f = 190 kHz). For each sample, multiple images from 5 × 5 μm were acquired at 3 different locations per disk. NanoScope Analysis 2.0 software were used to analyse the AFM images to obtain the Mean Roughness (Ra) and the Root Mean Square roughness (Rq). Ra and Rq were calculated and averaged from 3 to 5 identical scan areas (5 μm × 5 μm).

c) Scanning Electron Microscopy (FESEM) of material surface.

The surfaces of the **PDMS** and **PDMS-PIM** material was characterized by Field Emission Scanning Electron Microscopy (FESEM, JEOL JSM-7400E). Briefly, the material was washed with water followed by 70% ethanol and air dried. The dried disk was then coated with thin platinum film using high resolution sputter coater (JEOL, JFC-1600 Auto Fine Coater; coating conditions: 20 mA, 30 s) and viewed under SEM.

d) Mechanical property of the PDMS and PDMS-PIM material.

Tensile stress-strain experiments was carried out to characterize the mechanical strength of our PDMS and PDMS-PIM material. The mechanical property of the PDMS materials were evaluated on 3340 Series Single Column Table Frames (INSTRON 3344 universal testing machine (Instron, Norwood, Massachusetts). The 25 mm × 25 mm dimension PDMS material was clamped to expose 12 mm midsection of the material. The test samples were mounted on two mechanical grips with one grip attached to the cross head where the load cell is mounted and other grip attached to the fixed end. The initial length of the sample was recorded and subsequently the device stretched the samples, vertically at a constant speed of 10 mm/min. Data was collected and processed using the respective software. All tests were conducted at room temperature and at least in duplicates for each tested PDMS material (pristine and exposed to microbes). Relationship between the stress and strain, load (N), ultimate tensile strength (MPa), elongation (mm) and modulus (MPa) were determined at end point at maximum elongation.

### Characterization of PIM45-vinyl released in solution

The stability of the antimicrobial compound in the casted composite **PDMS** material was evaluated by both quantitative and qualitative assessments. All experiments were carried out in at least three replicates.

- The identity of the compound eluted from the **PDMS-PIM** material was confirmed by ^1^H NMR Spectroscopy. Composite **PDMS-PIM** material (1 cm^2^) was immersed in D_2_O and incubated at 300 rpm/room temperature for 1 h. ^1^H NMR spectroscopy was then carried out on the D_2_O solution to determine the chemical profile of the released antimicrobial compounds.
- The concentration of released **PIM45-vinyl** was determined using spectrophotometric method. UV spectrum scan was carried out for **PIM45-vinyl** in solution. Maximum absorption (λmax) at 252 nm was observed for the polymer and subsequently used for the semi-quantitative assay. Briefly, the biomaterial (100 mg) was placed in 10 ml of phosphate-buffered saline (PBS) in 15 ml centrifuge tubes and incubated at 300 rpm at room temperature for 1 h. The **PDMS** material was sequentially and periodically transferred to a fresh tube containing 10 ml PBS. The resultant PBS solution containing the released antimicrobial was subsequently used for semi-quantitative assay.
- Antimicrobial activity of the eluates were determined using the GB 15979–2002 protocol and as described by Malcolm et al. [22] Briefly, 10 ml PBS containing the released antimicrobial compounds (from 100 mg of **PDMS-PIM** material) was inoculated with microbes (bacteria, *E. coli* and *S. aureus* or fungi, *C. albicans*) to a final concentration of 10^4^ CFU/mL. The inoculum was incubated at room temperature for 1 h at 100 rpm. The bactericidal and fungicidal efficiencies of the leached compounds were evaluated by the plate assay. The viable colonies was computed for colony forming units per milliliter (CFU/mL) of inoculum.

### Surface antimicrobial assay

The antimicrobial property of the fabricated biomaterial was determined using the Japanese Industrial Standard (JIS Z 2801: 2010) Protocol [23]. Briefly, 100 ul microbial in suspension at 10^6^ CFU/mL in pure media (TSB or YMB; unless otherwise specified), was applied onto the surface of pristine **PDMS** or **PDMS-PIM** material (2.5 cm × 2.5 cm) disk. A thin plastic film of 2 cm × 2 cm was then overlaid on the cell suspension. The disk was incubated at appropriate temperature for 24 h (standard protocol). Incubation time point was adjusted as deviation from the standard protocol (for end point assays) as deem necessary. The material was then rinsed with 9.9 ml of TSB/YMB and the cell suspension was plated on LB agar plates. The plates were incubated at 36 °C for 16–18 h and viable microbe indicative of colony forming units (CFU/mL) was computed. Representative microbes, Gram negative (*E. coli*), Gram positive (*S. aureus*) and fungi (*C. albicans*) have been used for the evaluation. All experiments were carried out in three replicates.

### Biofilm formation and anti-biofilm assay (time–kill study)

Biofilm development was carried out according to Fisher et al. [14] and Merritt et al. [24] with slight modification. Microbes (100 ul) at 10^6^ CFU/ml in media were applied on sterile glass cover slips in six well plates. The microbial inoculate was allowed to grow and attach itself onto the cover slip for 72 h to form mature biofilm. The cover slips were then rinsed with PBS to remove unattached, planktonic bacterium, following the growth of biofilm. Formation of the biofilm was confirmed following crystal violet staining. The **PDMS-PIM** material (in triplicate) were then placed over the biofilm (on cover slips). Following incubation, the bacteria samples were harvested according to Mandakhalikar et al. [25] at intervals of 1 h, 2 h, 4 h and 24 h. Plate assay was carried out to determine colony forming units of viable bacteria. All experiments were carried out in three replicates.

### Long-term stability via continuous microbial challenge

The continuous microbial challenge protocol was adapted from Zhang et al. [26] with slight modifications. The experimental protocol is similar to the contact killing assay (JIS Z2801–2010 protocol) albeit with extended incubation period. Briefly, the **PDMS** and **PDMS-PIM** disk was sterilized via UV exposure for 30 mins. Microbial suspension at 10^6^ cells (100 ul for 2.5 cm × 2.5 cm disk) in respective media (TSB for *S. aureus* or YMB for *C. albicans* or both) was applied onto the **PDMS** and **PDMS-PIM** material and covered with thin plastic films (2 cm × 2 cm). The samples were incubated at 37 °C for stipulated time points. Bacterial suspension was periodically inoculated/refreshed every 48 h–72 h incubation and continued until the endpoint. At a pre-determined end-point (3 Days, 5 Days, 7 Days, 15 Days, 30 Days, 45 Days and 60 Days), the respective **PDMS** and **PDMS-PIM** materials were rinsed with 10 ml PBS and plated, following appropriate dilutions to compute for colony forming units (CFU/mL). All experiments were carried out in three replicates.

### Biocompatibilty evaluation of PDMS-PIM biomaterial

Both red blood cell hemolysis assay and mammalian cell viability assay was used to evaluate the biocompatibility of our **PDMS-PIM** material [27].

### Hemolysis assay

Fresh blood (mice) was rinsed 5 times in saline (0.9% NaCl) at 500 g/5 mins to remove the plasma. The subsequent red blood cells (RBC) was rinsed 2x in PBS to prepare the working solution. RBC suspension was diluted to 4% vol/vol in PBS for the hemolysis assay. The diluted RBC (100 ul) were added to 96 well plate followed by 100 ul PBS, in the presence of 5 mg or 10 mg **PDMS-PIM** material and incubated at 35 °C for 1 h. The plates were then centrifuged at 2200 rpm/5 mins. An aliquot (100 ul) of the supernatant was transferred to a new 96 well plate. The hemoglobin released upon RBC lysis was determined spectrophotometrically at 576 nm. The percentage of lysis was calculated using TritonX100 treated positive control and untreated control RBC [28]. The data was analyzed and expressed as mean and standard deviation of three replicates for quantification.

### Cell viability assay

Cell viability assay was carried out using the murine derived fibroblast (L929) in culture. L929 murine fibroblast cells were cultured in DMEM complete media (supplemented with 10% (v/v) heat-inactivated fetal bovine serum (FBS), 100 U/ml penicillin and 100 μg/ml streptomycin) at 37 °C in 100% humidity and 5% CO_2_. Fibroblast cells were seeded at 1.0 × 10^4^ cells/well (100 ul) in 96 well plate for overnight growth. The overnight culture of L929 in 96 well plate were incubated with 5 mg and 10 mg **PDMS** and **PDMS-PIM** material. Cell viability assay was carried out using the Alamar Blue reagent according to manufacturer’s protocol (Thermofisher Inc). Briefly 10 ul of Alamar Blue Reagent was added to 90 ul of complete DMEM media (DMEM containing 10% FBS and 1% Penicillin/Streptomycin) in the wells and incubated for 1–4 h at room temperature. Fluorescent intensity of viable cells were read at 570 nm/590 nm. The cell viability was calculated as the ratio of the absorbance of treated cells to the absorbance of the control groups (untreated). All experiments were performed in triplicate in three independent experiments.

### Statistical analysis

Data were expressed as means ± standard deviation of the mean (S.D.). Student’s *t*-test was used to determine significance among groups. A difference with *P* < 0.05 was considered statistically significant.* *p* < 0.05, ** *p* < 0.01, *** *p* < 0.001.

## Results

**PDMS** is the choice material for a wide range of medical applications, especially for catheter preparation. A number of strategies have been developed for **PDMS** surface modification to add on additional functions for specific applications. Here, we propose a bulk modification method to make antimicrobial **PDMS**-based biomaterials instead of device surface modification. With the new **PDMS** composite materials (**PDMS-PIMx**, x = weight percentage of **PIM** in composites), one can make any device or product based on **PDMS** with inherent disinfection and antifouling property.

**Scheme 1:**
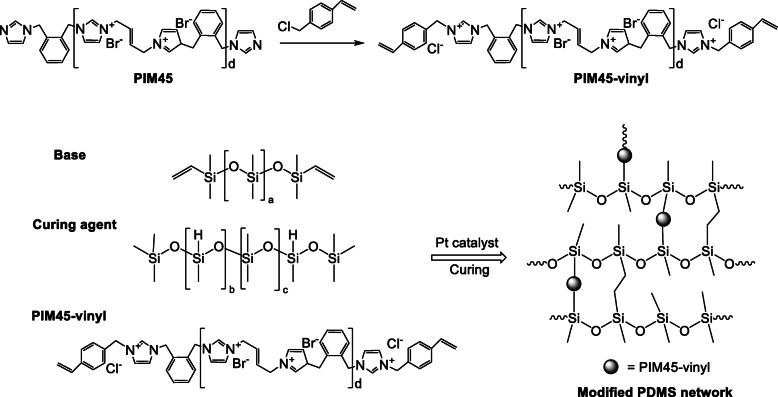
Preparation of antimicrobial **PDMS** composite biomaterial

Imidazolium polymer **PIM45** is reported with excellent antimicrobial efficacy and the polymer chains are typically ended with imidazole group [19, 20]. Platinum is a good catalyst for the hydrosilylation of styrene and its derivatives [29]. Herein, **PIM45-vinyl** (Scheme 1, Fig. S1) was casted with **PDMS** to fabricate a biomaterial (**PDMS-PIM**). In this study, THF was selected as solvent to mix **PIM45-vinyl** and **PDMS** base and curing agent homogenously to proceed the mix-cure process. The quasi-solid substance were degassed under vacuum to remove solvent and air bubbles followed by curing at 70 °C for overnight. **PDMS** biomaterials (**PDMS-PIM0.5, 1, 2, 5**) with different **PIM** loadings (0.5 wt%, 1 wt%, 2 wt% and 5 wt%) were synthesized.

To verify the chemical bonding of **PIM** component within the **PDMS** network, a leaching experiment was carried out for the synthesized **PDMS-PIM** materials. Materials including **PDMS-PIM1**, **PDMS-PIM2**, **PDMS-PIM5** and PDMS-PIM*1 (This sample is synthesized by using **PIM45** instead of **PIM45-vinyl**. It should not have chemical bonding between **PIM** component and **PDMS** network.) were immersed in PBS solution or deuterated H_2_O to assess if the **PIM** component is released from the **PDMS-PIM** material. **PIM** concentrations in PBS solution were calibrated and calculated by UV-vis spectroscopy (Fig. S2). **PIM** component in D_2_O was characterized by ^1^H NMR spectrum (Fig. S3).

**Fig. 1:**
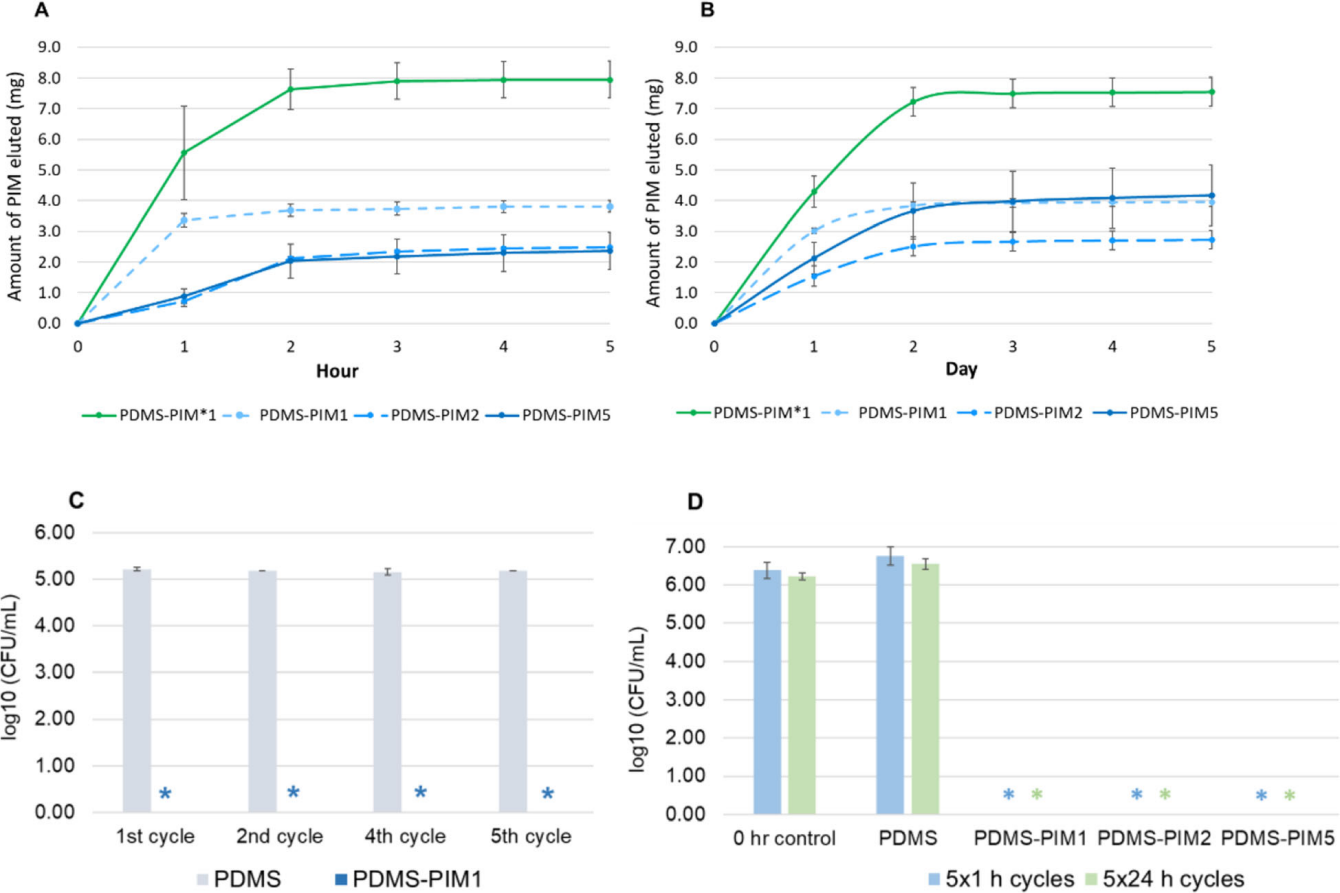
Evaluation of the control release of antimicrobial from composite biomaterial. Quantitation of imidazolium **PIM45** and **PIM45-vinyl** derivatives that was released/eluted in PBS over (**A**) 5 × 1 h cycles, (**B**) 5 × 1 day cycles. Quantitation was carried out using spectrophotometric assay based on calibration curves that were generated from the parent **PIM45-vinyl** compound (Fig. S2). The eluted compound in solution (**C**) and the composite material post elution of antimicrobial compound was subjected antimicrobial assay (**D**) against *S. aureus*. * denotes zero (0) CFU on the agar plate for undiluted samples

The release of **PIM** component from **PDMS-PIM** in PBS was observed over 5 cycles, indicating a sustained release of antimicrobial compound (Fig. 1A: 1 h per cycle and 1B: 1 day per cycle). Semi-quantitative assay further showed an initial burst of **PIM** component in the first two cycles followed by a gradual and sustained release up to five cycles (Fig. 1). A cumulative 5.6% of **PIM** component was released from the **PDMS-PIM5** material in contrast to 90% release for PDMS-PIM*1 material. It is clear that the **PDMS-PIM** materials synthesized with **PIM45-vinyl** have much slower release profiles as compared with the material synthesized with **PIM45** (without vinyl end groups) and this suggests that the chemical bonds were formed between **PIM** component and **PDMS** framework through hydrosilylation reaction (Fig. S4). The eluted solution and the recovered **PDMS-PIM** biomaterial (after multiple cycles of PIM elution) exhibit antimicrobial activity (Fig. 1C, D). Furthermore, D_2_O solutions in which **PDMS-PIM1** and **PDMS-PIM2** materials were submerged contained dissolved **PIM** component lacking vinyl groups, as characterized by ^1^H-NMR spectroscopy. A minor amount of **PIM** component released from **PDMS-PIM5** material contained the vinyl functional group (Fig. S3). These results indicate that the hydrosilylation reaction of **PIM45-vinyl** is complete and all the **PIM45** is chemically bonded in materials with 1 and 2 wt% **PIM45-vinyl** loading, but incomplete in materials with 5 wt% **PIM45-vinyl** loading. In **PDMS-PIM1** and **PDMS-PIM2** materials, the **PIM** leaching may result from hydrolysis of Si-O bonds in the **PDMS** framework, which was also verified by ^1^H-NMR spectroscopy (Figs. S4, S5).

Our observation confirms that modifying the end group of an antimicrobial compound can be used to fabricate composite biomaterials. The resulting composite material integrates and retains the antimicrobial component in a manner which allows gradual and sustained release of the antimicrobial compound.

**Fig. 2:**
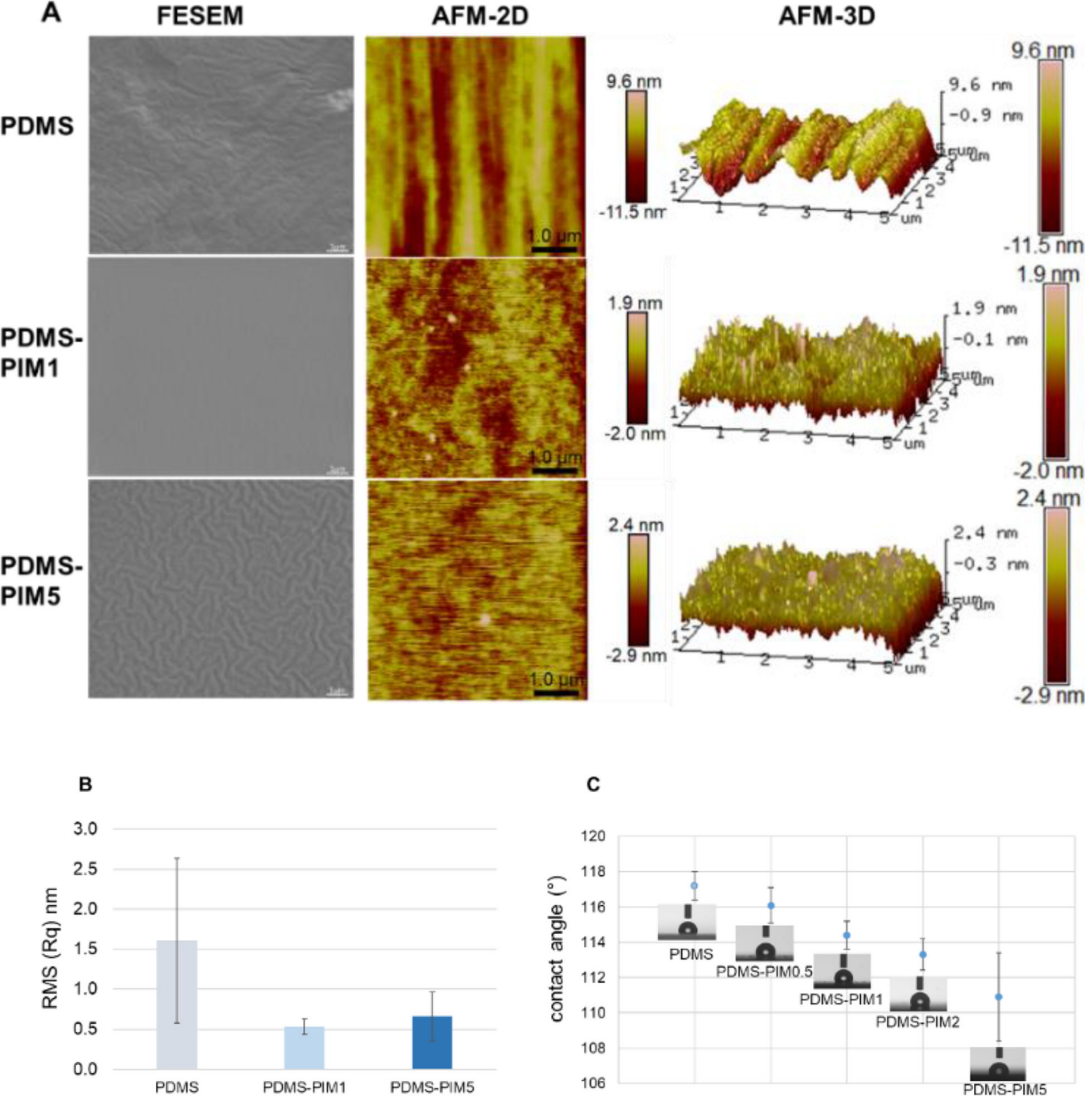
Surface characteristics of composite **PDMS-PIM**. (**A**) Surface topology of **PDMS** and **PDMS-PIM** material. Images of **PDMS** material surface on FESEM and AFM. (**B**) AFM analysis on surface roughness. Results are depicted as root mean square (RMS) values (Rq, nm) for respective unmodified **PDMS** and **PDMS-PIM** biomaterial. (**C**) Surface wettability analysis. Contact Angle measurement was carried out to determine the wetting ability of **PDMS** and **PDMS-PIM** material surface. The results are expressed as mean and standard deviation of 3 × 3 replicates

Surface topology of the biomaterial was determined using both FESEM and the AFM methodology. Surface roughness analysis on AFM showed that the root mean square (Rq) of the **PDMS** surface becomes even smoother upon inclusion of **PIM45-vinyl** (Fig. 2). Data from AFM corroborates with the SEM images which show that the surface of **PDMS** and its **PDMS-PIM** materials (**PDMS-PIM1** and **PDMS-PIM5**) are smooth. The surface of **PDMS-PIM** materials are smooth and intrinsically hydrophobic. The contact angle measurements indicated that the surfaces of the **PDMS-PIM** biomaterials are generally less hydrophobic as compared with **PDMS** (Fig. 2C). Increasing the amount of integrated polymeric antimicrobial compound, which is hydrophilic in nature, decreases the hydrophobicity of the surface.

**Fig. 3:**
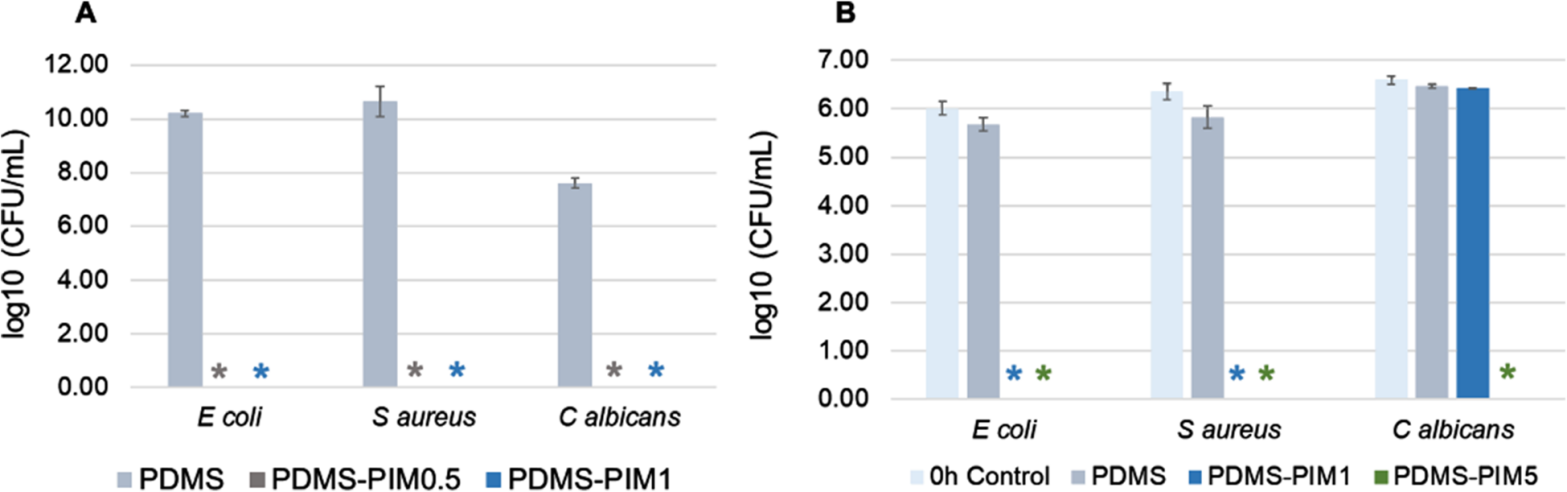
Surface antimicrobial assay for **PDMS** and **PDMS-PIM** materials (**PDMS-PIM0.5**, **PDMS-PIM1** and **PDMS-PIM5**). Materials have been stored at room temperature for up to 6 months. (**A**) Surface antimicrobial assay was carried out using representative of Gram negative (*E. coli* #15036); Gram positive (*S. aureus* #6538) and Fungi (*C. albicans* #10231) microbes following the JIS Z 2801–2010 standard protocol at 24 h time point using neat/pure media. (**B**) Rapid killing efficacy of **PDMS-PIM** material was carried out based on JIS Z 2801 standard protocol with 30 s exposure of microbes. * denotes zero (0) CFU on the agar plate for undiluted samples. The results are expressed as mean and standard deviation of three replicates

The surface antimicrobial property of the fabricated biomaterial was evaluated using modified Japanese Industrial Standard protocol [23]. The microbes (in neat media) were exposed to composite **PDMS-PIM** for 24 h. The data revealed that **PDMS-PIM0.5** and **PDMS-PIM1** have potent biocidal activity against *E. coli, S. aureus* and *C. albicans* (Fig. 3A). The time killing assay performed with **PDMS-PIM1** and **PDMS-PIM5** to assess the shortest exposure time needed to eliminate microbes on the surface of **PDMS** material. We found that the bactericidal property of **PDMS-PIM1** was effective within 30 s of microbial exposure (Fig. 3B), exhibiting complete eradication of the microbes (single inoculation). In addition, **PDMS-PIM** materials used in this test have been stored at room temperature for up to 6 months.

**Fig. 4:**
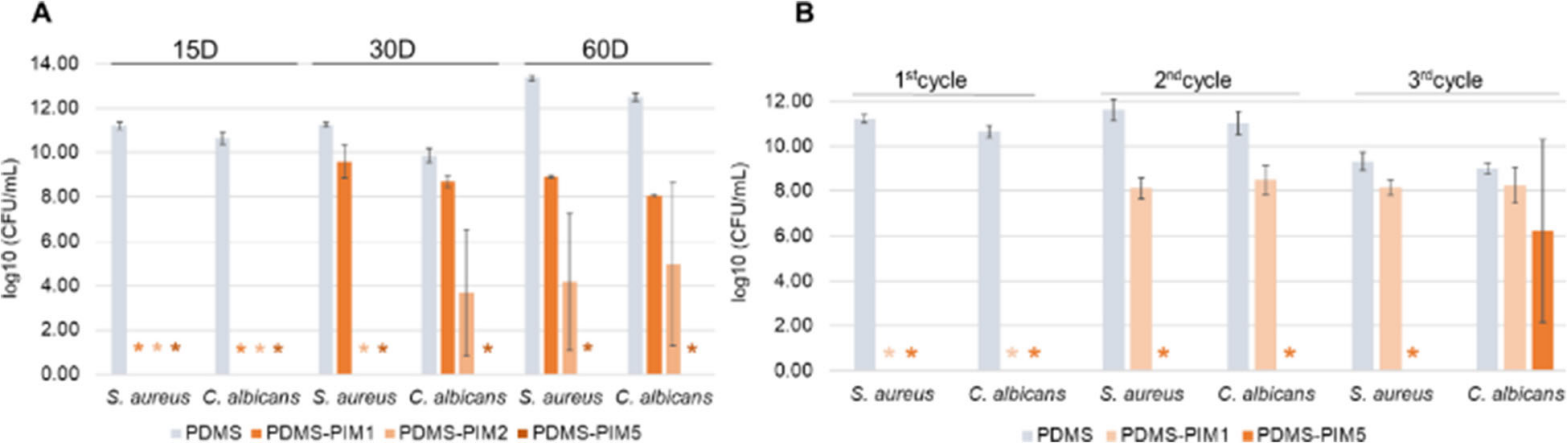
(**A**) Durability of **PDMS-PIM** biomaterial in microbial co-culture with continuous challenge –inoculation at 48–72 h interval. The biomaterial was challenged with microbes (*S. aureus* and *C. albicans*) at 48–72 h intervals. The antimicrobial potency evaluation was carried out at 15 days, 30 days and 60 days endpoint. (**B**) Recycle/Reusable potential of recovered **PDMS-PIM** disk. Each cycle indicates a 15 days period that the biomaterials were challenged with microbial at 48–72 h interval, periodically, before being assessed for the antimicrobial potency. The results are expressed as mean and standard deviation of three replicates. * indicates zero (0) CFU on the agar plate for undiluted samples

The surface antimicrobial assay was repeated over longer durations to study the stability and durability of the **PDMS-PIM** biomaterial [26, 30]. The antimicrobial **PDMS-PIM** disks were challenged with respective microbes every 48–72 h. The study was terminated at 45 days for single culture while three time points at 15 days, 30 days and 60 days were recorded for co-culture assay. In single culture assay, we observed complete bactericidal effect up to 45 days for **PDMS-PIM** biomaterials against both *S. aureus* and *C. albicans.* In contrast, the unmodified **PDMS** biomaterial exhibited colonization at 10^11^ CFU/mL and 10^10^ CFU/mL for *S. aureus* and *C. albicans* respectively (Table S1). Since multi microorganism infections and colonization is widespread in healthcare settings, we initiated a *S. aureus* and *C. albicans* co-culture in the laboratory and tested it on the composite **PDMS-PIM** materials (Fig. 4A). Microbes in this co-culture was identified by the phenotypic characteristics of the colonies. *S. aureus* forms golden colored colonies that distinguishes from the white colonies formed by *C. albicans*. **PDMS-PIM1**, **PDMS-PIM2** and **PDMS-PIM5** materials could fully eradicate co-cultured bacterial and fungal colonization after 15 days in culture*.* At the 30 day time point, reduction in antimicrobial potency was observed for **PDMS-PIM1** against both *S. aureus* and *C. albicans.* While **PDMS-PIM2** exhibited moderate efficacy against *C. albicans* but fully eradicated *S. aureus* (Fig. 4 A), a dose dependent phenomena. It is noteworthy that **PDMS-PIM5** eliminated both *S. aureus* and *C. albicans* even after 60 days in co-culture, demonstrating excellent durability. Such qualities make **PDMS-PIM5** suited for practical medical applications, especially as long-term indwelling urinary catheters which typically could remain in place for more than 30 days.

To evaluate the reusability of the composite biomaterial, the **PDMS-PIM** materials recovered on Day 15 of co-culture incubation were subjected to a further 2 cycles (15 days / cycle) of co-culture incubation (Fig. 4B). We found that **PDMS-PIM5** could last for 3 cycles of 15 days each (45 days in total) with washing and rinsing carried out every 15th day. Thus, **PDMS-PIM5** exhibited stable antimicrobial properties after 3 cycles of periodical rinsing or washing. Reuse of the recovered **PDMS-PIM** material also confirmed that **PDMS-PIM5** could effectively eradicate colonization of bacteria in co-cultures.

In addition, the mechanical strength and mechanical properties of the material have been evaluated for the potential use as a catheter. Tensile strength/stress testing experiments indicate that the mechanical property of the PDMS material was not altered with incorporation of PIM45-vinyl (Table 1). Furthermore, the data also shows that the tensile strength remained unaltered with the extended durability. It is noteworthy that the tested material did not break/fracture during the experimental procedures [14, 31]. Statistical analysis (*t*-test, 2 tailed homoscedatic) Student’s *t*-test between control and composite materials (pristine or used for either 45 Days or 60 Days) and with regards to load, tensile strength and modulus is shown in Table S2.

**Table 1:** Tensile strength and modulus of PDMS materials

Materials	Modulus (Automatic Young’s) [MPa]	Maximum Force [N]	Tensile stress at Maximum Force [MPa]
**As synthesized**			
**PDMS**	1.78 ± 0.35	13.83 ± 1.94	0.51 ± 0.07
**PDMS-PIM1**	1.38 ± 0.38	10.30 ± 2.34	0.37 ± 0.11
**PDMS-PIM5**	1.11 ± 0.28	10.39 ± 1.55	0.37 ± 0.04
**45 Days of Microbial Exposure**			
**PDMS**	1.79 ± 0.06	14.13 ± 4.20	0.43 ± 0.13
**PDMS-PIM1**	1.78 ± 0.25	11.73 ± 1.73	0.36 ± 0.06
**PDMS-PIM5**	1.31 ± 0.20	13.34 ± 2.64	0.42 ± 0.07
**60 Days of Microbial Exposure**			
**PDMS**	1.91 ± 0.02	14.83 ± 2.06	0.40 ± 0.05
**PDMS-PIM1**	1.76 ± 0.45	11.24 ± 1.06	0.39 ± 0.03
**PDMS-PIM5**	1.28 ± 0.09	10.27 ± 2.28	0.39 ± 0.03

**Fig. 5:**
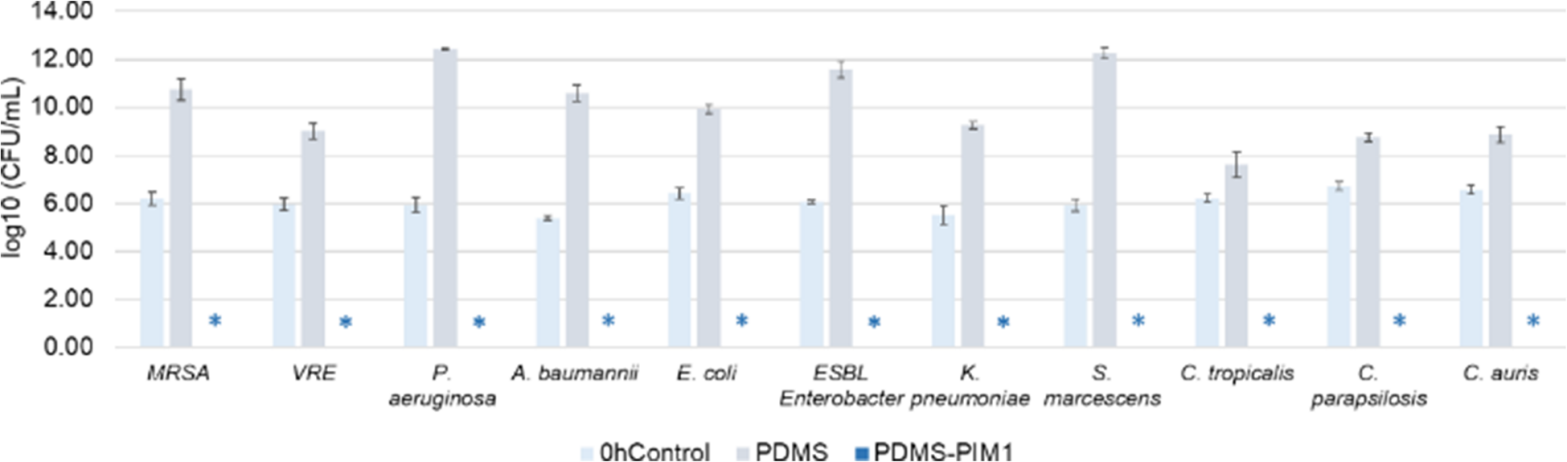
Efficacy of antimicrobial activity of **PDMS-PIM1** on clinical pathogens. Potency of polymer antimicrobial **PDMS-PIM1** against clinical pathogens (*Enterococcus faecium-VR Enterococcus; Staphylococcus aureus-MRSA; Klebsiella pneumoniae, Acinetobacter baumannii, Pseudomonas aeruginosa, Enterobacter species-ESBL Enterobacter, S. marcescens, E. coli*, *C. parapsilosis*, *C. tropicalis and C. auris*) was evaluated following JIS Z 2801–2010 protocol. The results are expressed as mean and standard deviation of three replicates. * denotes zero (0) CFU on the agar plate for undiluted samples

In order to assess the potential value of our biomaterial in medical device fabrication, the biomaterial was tested for its antimicrobial efficacy on clinical isolates that are prevalent in hospital acquired infections (HAI). These microbes represent various antibiotic resistant (MDR) species often related to nosocomial infections in healthcare settings, especially in CAUTI. We found that **PDMS-PIM1** inhibited the growth of all the clinical isolates. These microbes represent various MDR species often related to nosocomial infections in healthcare settings, especially in CAUTI. The acronym **ESKAPE** comprise of six nosocomial pathogens that exhibit multidrug resistance and virulence, namely *Enterococcus faecium, Staphylococcus aureus, Klebsiella pneumoniae, Acinetobacter baumannii, Pseudomonas aeruginosa*, and *Enterobacter* spp. Continual use of antibiotics has aggravated the emergence of MDR and extensively drug resistant (XDR) bacteria, that render even the most effective drugs ineffective. Extended spectrum β-lactamase (ESBL) and carbapenemase producing Gram negative bacteria have emerged as a recalcitrant therapeutic challenge. These MDR microbes are the most notorious group of bacteria that pose a serious global health threat [32, 33]. We found that our antimicrobial biomaterials (**PDMS-PIM1**) inhibited the growth of all the **ESKAPE** group of clinical isolates as well as **MRSA** and Vancomycin-resistant *Enterococcus* (**VRE**) pathogens (Fig. 5) and respective *Candida sp* of clinical isolates.

**Fig. 6:**
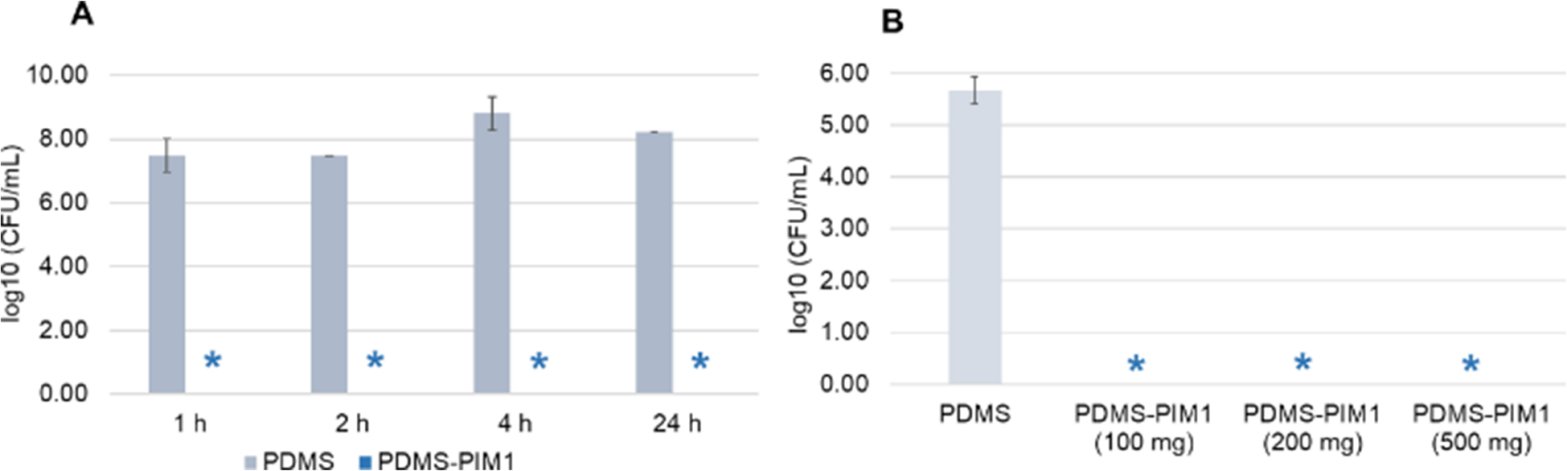
Eradication of biofilm by **PDMS-PIM1**. (**A**) Biofilms were formed on glass cover slips. **PDMS** and **PDMS-PIM1** material were overlaid on the biofilm and incubated at 35 °C for respective time points. The biofilm bacteria inoculate was harvested in 10 ml PBS and plated on LB agar plates following appropriate dilution. (**B**) **PDMS** material and **PDMS-PIM1** material were immersed in 10 ml PBS. Tubes (2 cm × 3) with preformed biofilm in lumen were then incubated in the presence of **PDMS-PIM** material of varying weights, for 1 h at 100 rpm/35 °C. The viable colonies were determined by plate assay in both experiments. The results are expressed as mean and standard deviation of three replicates. * denotes zero (0) CFU on the agar plate for undiluted samples

Microbial colonization and formation of biofilm have become the major threat that leads to emergence of drug resistant microbes. Often, antimicrobial resistance is enhanced by the colonization of microbes and formation of biofilms. We formed biofilms on polystyrene, **PDMS**, low density polyethylene (tube) and glass cover slips and subsequently exposed these biofilms to **PDMS-PIM1** material [14, 25, 34]. We found that **PDMS-PIM1** could effectively eradicate biofilm colonies on all the surfaces. A time dependent assay showed that biofilm colonies on glass cover slips were eradicated within 1 h of contact with **PDMS-PIM** composite material (Fig. 6A). Biofilm formed in a tube could be eliminated by the eluate from 100 mg of **PDMS-PIM1** material in 10 ml PBS solution (Fig. 6B) [22].

Microbial adhesion onto a surface is the initial stage of any pathogenesis, which subsequently leads to formation of the difficult to treat biofilms. **PDMS**, being inherently hydrophobic, exhibits an inherent antifouling property as a biomaterial as it does not support adherence of microbes to its surface. Inclusion of the **PIM** antimicrobial compound further enhances the antifouling and subsequently biocidal/antibiofilm properties of the **PDMS-PIM** material, thus fulfilling the desired properties of an antimicrobial biomaterial.

**Fig. 7:**
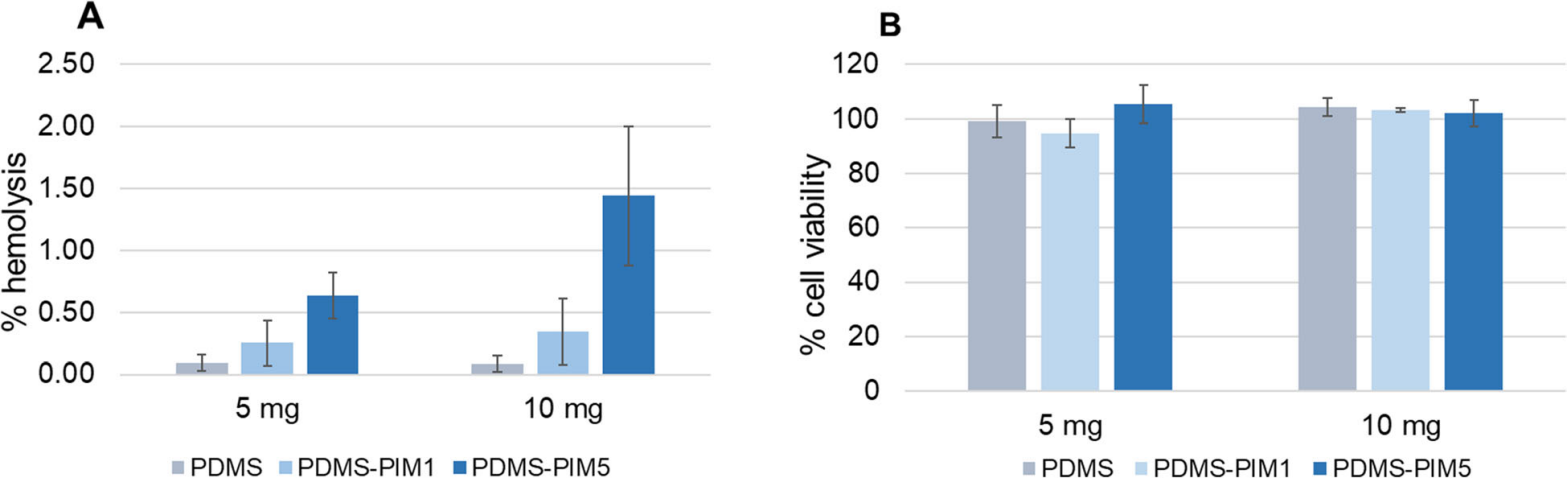
Biocompatibility analysis. (**A**) Hemocompatibility assessment was carried out using red blood cell hemolysis assay. **PDMS-PIM** biomaterial (5 mg and 10 mg) in 100 μl of red blood cells was incubated at 35 °C at 150 rpm for 1 h. The lysis of RBC was determined spectrometrically at 576 nm. (**B**) Cytocompatibility evaluation was carried out using L929 fibroblast cell viability assay. **PDMS-PIM** biomaterial (5 mg and 10 mg) was added to the L929 cells (in 96 well plate) in 100 μl of DMEM complete media. The plate was incubated at 35 °C with 5% CO_2_ for 24 h. Cell viability was determined using the Alamar Blue reagent. The results are expressed as mean and standard deviation of three replicates

Assessment of the materials’ biocompatibility, comprising hemocompatibility and cytocompatibility assays, [27, 28] was carried out to evaluate the safety of **PDMS-PIM** biomaterial for potential medical application. A 10 mg sample of **PDMS-PIM5** material exhibited 1.44 ± 0.56% hemolysis, while **PDMS-PIM1** showed 0.3 ± 0.26% hemolysis (Fig. 7A). Cell viability assay using murine derived fibroblast L929 cells (Fig. 7B) also proved that our composite biomaterial is non-toxic to mammalian cells in culture. Hence, we found that the **PDMS-PIM** materials are biocompatible supported by both the hemo- and cyto-compatibility assessments.

## Discussions

Catheter associated infections (CAI) pose a serious health threat and remains a challenge in healthcare settings worldwide. Patients requiring indwelling catheters, especially urinary catheters, often get infections within days of catheter use. Though antimicrobial-coated catheters are currently available, their efficacy in clinics are still far from satisfactory [6–8]. In this study, **PDMS** based biomaterial containing an antimicrobial imidazolium polymer demonstrated several key characteristics which may indicate the potential application of these materials to mitigate catheter associated infections. **(1) PDMS-PIM** biomaterial exhibited potent antimicrobial activity and fast killing property with 30 s of exposure to the microbes resulting in complete eradication of the microbes. The material demonstrated long lasting activity that could inhibit the colonization of both *S. aureus* and *C. albicans* for at least 45 days in culture. Eradication of microbial colonization on a subsequent inoculation of the recovered and reused material further confirm that our **PDMS-PIM** material could be recycled for usage. The **PDMS-PIM** material does not cause any change or adverse effect on the mechanical performance of the fabricated materials, either pristine or used products. The tensile property of the fabricated materials were also not altered with extended use, thus confirming their durability. **(2)** Controlled release of antimicrobial substance from a polymeric material has been shown to be useful in preventing planktonic as well as biofilm microbial growth on biomaterials [34–38]. It is a challenge to control the amount of antimicrobial compound that is coated, embedded or impregnated from the outset. In contrast, the method described herein results in a composite **PDMS-PIM** biomaterial casted with a well-defined amount of antimicrobial polymer that is fully integrated and chemically bonded in the material. The amount of antimicrobial compound in the biomaterials can be accurately tuned to fit the requirements and usage conditions of the resulting device. **(3) PDMS-PIM** materials continues to release active compounds over a prolonged period of time. The releasing process mainly involves hydrolysis of Si-O bonds. Initial burst release was followed by subsequent sustained release of active antimicrobial compound. The release profile is demanded to curtail the biofilm formation and sustain continuous treatment [39]. **(4)** The **ESKAPE** family of MDR bacteria are the most notorious group of bacteria that pose a serious global health threat and account for up to 87% of all hospital acquired infections [40]. This family of bacteria has been listed as “high priority” under the WHO priority listing [41–45]. It is noteworthy that **PDMS-PIM** biomaterials could efficiently and effectively inhibit the colonization of all the members of **ESKAPE** group of pathogens as well as the WHO priority pathogens such **MRSA** and **VRE**. The susceptibility of these drug resistant bacteria towards **PDMS-PIM** further endorse that our composite biomaterials in the form of catheters could prove beneficial in preventing catheter-associated infections especially in CAUTI. In addition, the composite **PDMS-PIM** biomaterial is biocompatible and exhibits stable antimicrobial activity up to at least 6 months storage at room temperature.

## Conclusions

We have fabricated a biocidal **PDMS-PIM** biomaterial that shows sustained release of the polymeric antimicrobial. Our approach will prove beneficial in fabricating antimicrobial biomaterials for manufacturing devices for medical and healthcare applications. Compared to available products that rely on coating or impregnation to introduce antimicrobial components, the **PDMS-PIM** biomaterial presented in this study will be easier to manufacture. No additional steps to coat or impregnate the material with antimicrobial compounds are needed. In addition, the amount of antimicrobial compounds to be incorporated for fabrication can be regulated during the one-time casting process. The new material demonstrated excellent antimicrobial activity, being able to efficiently inhibit or eliminate the colonization of both Gram positive (*S. aureus*) and Gram negative (*E. coli*) bacteria as well as fungi (*C. albicans*) in single or co-culture experiments. Furthermore, **PDMS-PIM** composite material effectively functioned as a bactericidal and fungicidal surface against multidrug resistant clinical isolates including the **ESKAPE** family of pathogens. Stability and durability studies showed that the new material could withstand prolonged incubation with continuous challenge with microbes - up to 60 days against single culture bacterial or fungi or their co-culture. Hence, the **PDMS-PIM** materials have the potential to be used for antifouling/antibiofilm urinary catheters that require long-term continuous utilization*.*

## Supplementary Information

**Additional file 1.**

## Data Availability

All data are available on request.
