# Supplementary material for: Broad spectrum antimicrobial PDMS-based biomaterial for catheter fabrication
Source: Biomater Res. 2021 Oct 21;25:33. doi: 10.1186/s40824-021-00235-5 (PMC8529379; doi:10.1186/s40824-021-00235-5)
Supplement: Supplementary file 1 — Additional file 1. [file 40824_2021_235_MOESM1_ESM.docx]

**Supplementary Information**

**Broad spectrum antimicrobial PDMS-based biomaterial for catheter fabrication.**

Arunmozhiarasi Armugam,* Siew Ping Teong, Diane S. W. Lim, Shook Pui Chan, Guangshun Yi, Dionis Si Ying Yew, Cyrus W. Beh and Yugen Zhang*

Institute of Bioengineering and Bioimaging, 31 Biopolis Way, The Nanos, Singapore 138669, Singapore; email: [ygzhang@ibb.a-star.edu.sg](mailto:ygzhang@ibb.a-star.edu.sg)

**Supplementary Table**

**Table S1: Durability of PDMS-PIM biomaterial.** Single culture microbial inoculation with continuous challenge at 48-72 h interval, periodically, up to 45 Days in culture was conducted to evaluate the durability of the composite material.


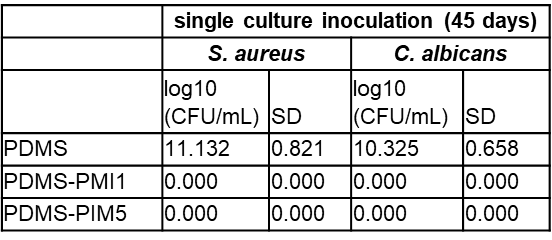


**Table S2. Statistical analysis (t-test, 2 tailed homoscedatic)**. Student’s t-test between control and composite materials (pristine or used for either 45 Days or 60 Days).

| **Modulus (Automatic Young's) [MPa]** | | | | | | | |
| --- | --- | --- | --- | --- | --- | --- | --- |
| **t test p value (2,2)** |  |  |  |  | **t test p value (1,1)** |  |  |
|  | **pristine** | **45Days** | **60Days** |  |  | **0-45 Days** | **0-60 Days** |
| **PDMS-PIM1** | *0.833* | *0.955* | *0.746* |  | **PDMS-PDMS** | *0.297* | *0.102* |
| **PDMS-PIM5** | *0.075* | *0.011* | *0.010* |  | **PIM1-PIM1** | *0.396* | *0.447* |
| **PIM1-PIM5** | *0.167* | *0.1011* | *0.407* |  | **PIM5-PIM5** | *0.185* | *0.5* |
| **Maximum Force [N]** | | | | | | | |
| **t test p value (2,2)** |  |  |  |  | **t test p value (1,1)** |  |  |
|  | **pristine** | **45Days** | **60Days** |  |  | **0-45 Days** | **0-60 Days** |
| **PDMS-PIM1** | *0.200* | *0.784* | *0.216* |  | **PDMS-PDMS** | *0.466* | *0.371* |
| **PDMS-PIM5** | *0.075* | *0.777* | *0.320* |  | **PIM1-PIM1** | *0.464* | *0.436* |
| **PIM1-PIM5** | *0.937* | *0.822* | *0.409* |  | **PIM5-PIM5** | *0.152* | *0.319* |
| **Tensile stress at Maximum Force [MPa]** | | | | | | | |
| **t test p value (2,2)** |  |  |  |  | **t test p value (1,1)** |  |  |
|  | **pristine** | **45Days** | **60Days** |  |  | **0-45 Days** | **0-60 Days** |
| **PDMS-PIM1** | *0.278* | *0.902* | *0.864* |  | **PDMS-PDMS** | *0.239* | *0.227* |
| **PDMS-PIM5** | *0.0338* | *0.930* | *0.776* |  | **PIM1-PIM1** | *0.410* | *0.471* |
| **PIM1-PIM5** | *0.975* | *0.483* | *0.451* |  | **PIM5-PIM5** | *0.285* | *0.398* |

**Supplementary Figures**


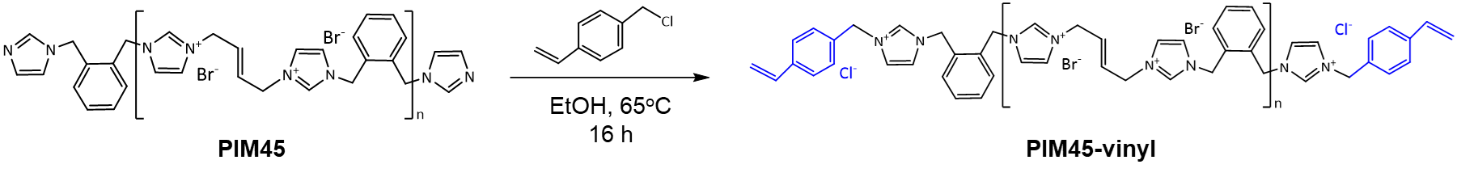


**Figure S1: The synthesis route for PIM45-vinyl.** The synthesis of **PIM45-vinyl** was carried out between **PIM45** with imidazole terminal and 4-vinylbenzyl chloride in the presence of ethanol at 65 ^o^C for 16 hours. The compound was purified from the suspension by repetitive precipitation with THF and dried under reduced pressure at 90 ^o^C.


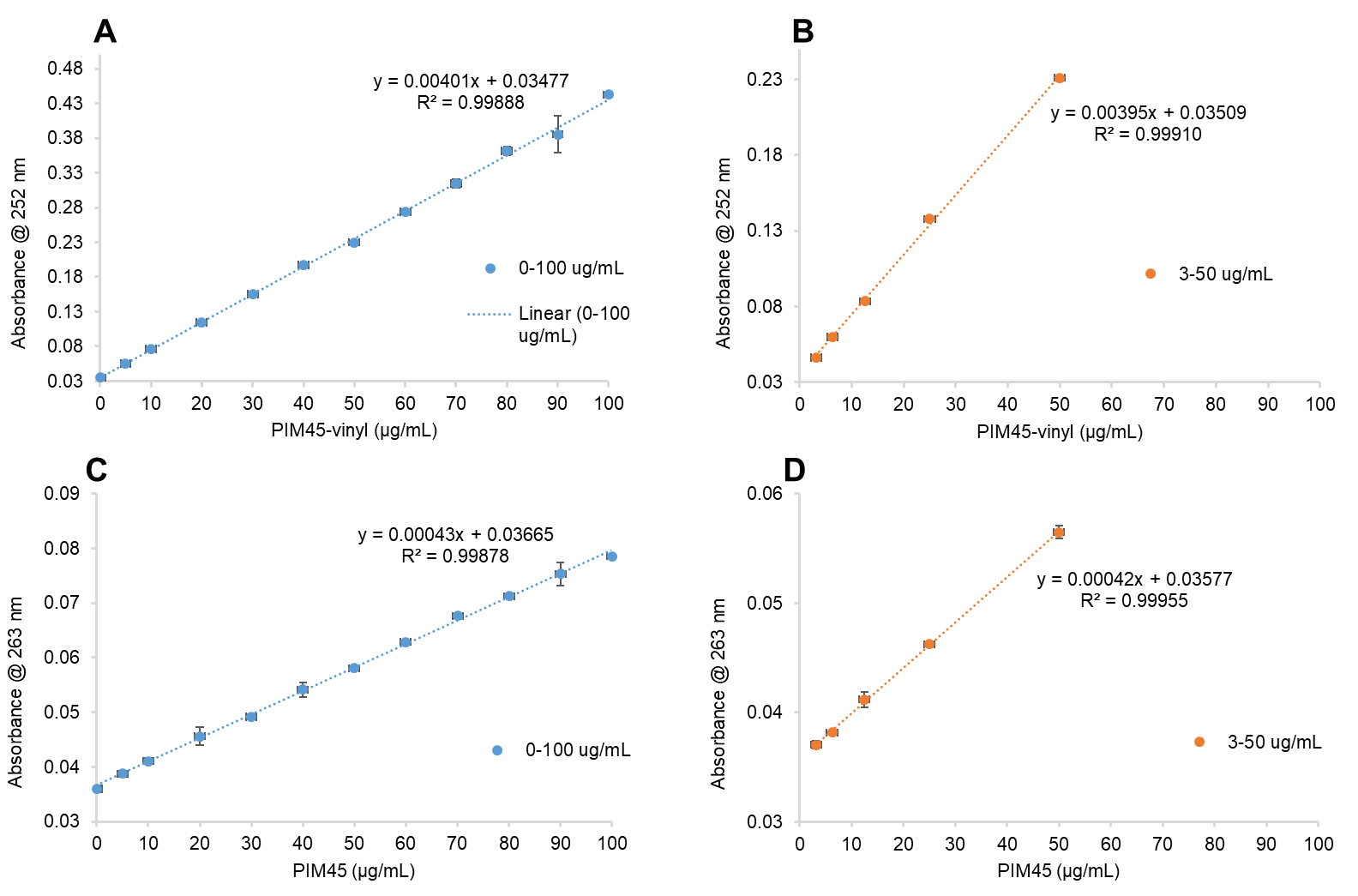


**Figure S2: Calibration curves for determination of PIM45-vinyl and PIM45 concentrations in PBS.** UV spectrum scan was carried out on the elution product to determine the λmax for the antimicrobials in PBS and subsequently calibration curves that follows Beer-Lambert Law were constructed for **(A)** 0-100 ug/ml **(**B) 63-1000 ug/ml **PIM45-vinyl**; and **(C)** 0-100 ug/ml **(D)** 3-50ug/ml parent PIM45 compounds in PBS, respectively.


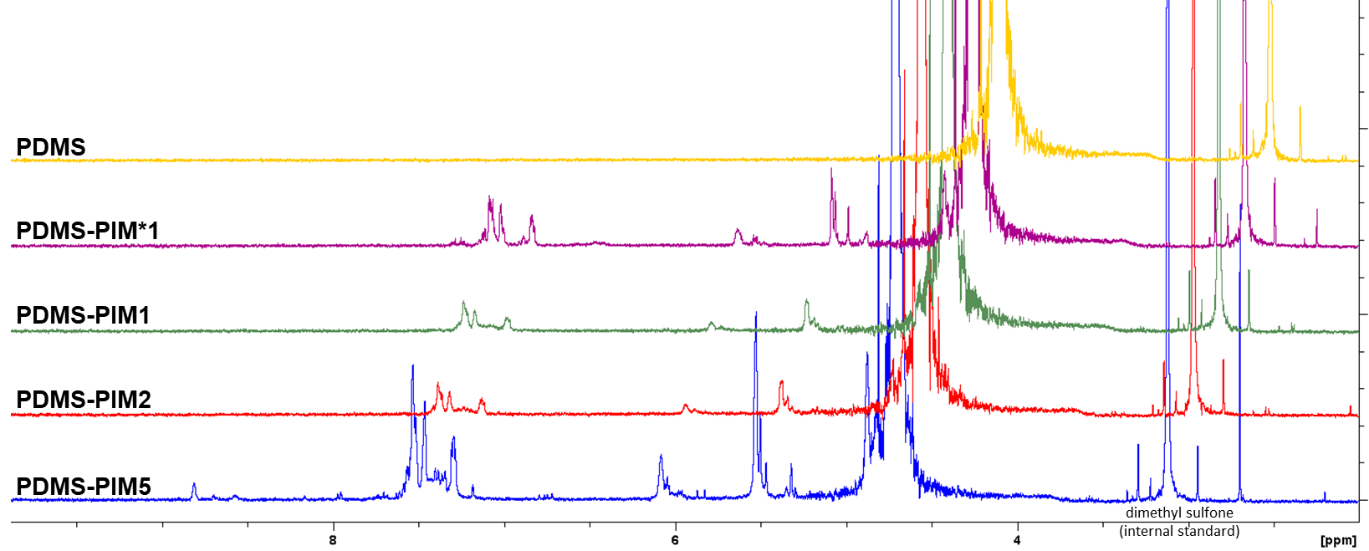


**Figure S3:** ^1^H-NMR spectrum of antimicrobial compound (PIM) released from **PDMS-PIM**. Biomaterial (100 mg) in 1ml of D_2_O was incubated at room temperature on shaking incubator (at 300 rpm). The D_2_O solution with containing the released active compounds were subjected to ^1^H NMR spectroscopy. A minute amount of **PIM** component released from **PDMS-PIM5** showed the presence of vinyl group (expected chemical shifts 6.75 ppm (doublet of doublets) & 5.82 ppm (doublet)). Dimethyl sulfone was used as internal control.


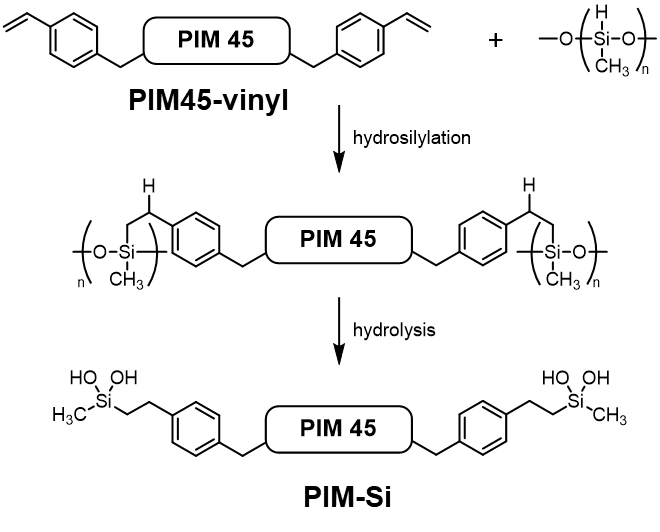


**Figure S4.** Scheme of hydrosilylation reaction and hydrolysis reaction.


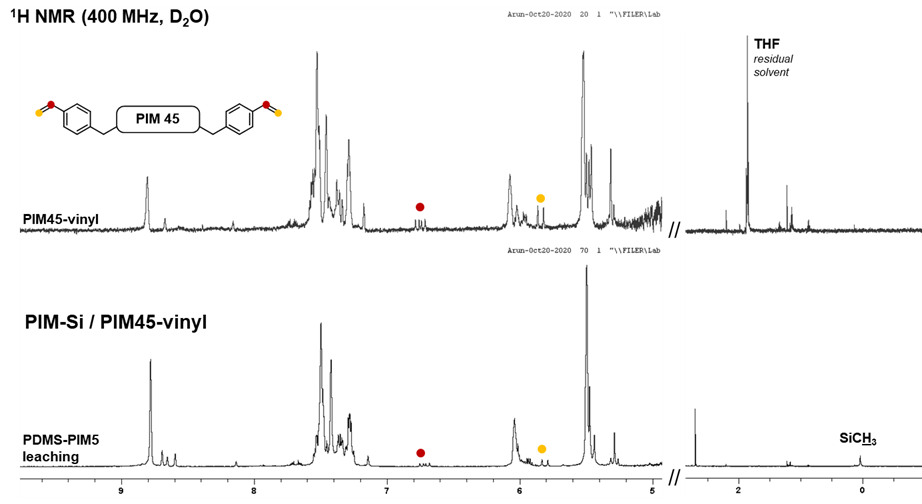


**Figure S5:** ^1^H NMR spectra of **PIM45-vinyl** and solution released from **PDMS-PIM5**, compounds proposed to be **PIM-Si** (Figure S4) and **PIM45-vinyl**.
